# Anti-rheumatic treatment and prosthetic joint infection: an observational study in 494 elective hip and knee arthroplasties

**DOI:** 10.1186/s12891-020-03459-z

**Published:** 2020-06-29

**Authors:** Ylva Borgas, Anders Gülfe, Mikael Kindt, Anna Stefánsdóttir

**Affiliations:** 1 Department of Rheumatology, Skåne University Hospital, SE-221 85 Lund, Sweden; 2 Department of Clinical Sciences, Section of Rheumatology, Lund University, Lund, Sweden; 3 Department of Orthopaedics, Skåne University Hospital, SE-221 85 Lund, Sweden; 4 Department of Clinical Sciences, Section of Orthopaedics, Lund University, Lund, Sweden

**Keywords:** Infections, Orthopedic surgery, bDMARD, Anti-TNF

## Abstract

**Background:**

Surgical site infections are more frequent among patients with rheumatic disease. To what extent this is related to immunosuppressive antirheumatic drugs is unclear, as is the value of discontinuing medication perioperatively. The aim of study was to assess the rate of surgical site infections after knee and hip replacement in patients with inflammatory joint disease, with an emphasis on periprosthetic joint infection, and to investigate the influence of treatment with disease-modifying antirheumatic drugs (DMARDs) in this regard.

**Methods:**

Data were collected from 494 primary elective hip (51.4%) and knee arthroplasties, along with demographic and medication data. The primary outcome was surgical site infection during the first year after surgery.

**Results:**

In 78% (*n* = 385) of the cases the patient used 1 to 3 disease-modifying antirheumatic drugs perioperatively. Thirty-two percent (*n* = 157) of patients used a TNF-alpha inhibitor. The rate of surgical site infection was 3.8% (*n* = 19). The rate of periprosthetic joint infection was 1.4% (*n* = 7), all of which occurred after knee arthroplasty. Periprosthetic joint infection occurred in only 1 patient medicating perioperatively with a TNF-alpha inhibitor.

**Conclusion:**

Surgical site infections were not associated with ongoing medication with disease-modifying antirheumatic drugs. Due to the low event rate this should be interpreted with caution, but our center will maintain its routine of continuing treatment with TNF-alpha inhibitors perioperatively.

## Background

In most affluent countries during the past 20 years, treatment with biologic disease-modifying drugs (bDMARDs) such as TNF-alpha inhibitors has become part of the standard of care for patients with rheumatoid arthritis (RA), as well as for other types of inflammatory joint disease. Although the need for joint arthroplasty in these patients has decreased [1–7], a number of patients still are admitted to surgery. Many of these patients are treated with conventional disease-modifying drugs (cDMARDs) and/or bDMARDs prior to the operation.

The incidence of infections in general is higher in patients with RA than in non-RA subjects. It is still not fully understood whether this is a consequence of immunologic disturbances due to the disease itself - as disease severity is a risk factor for infection - or of the immunosuppressive treatment often used.

TNF-alpha inhibitors are thought to increase the general risk of infection [8, 9].

Studies vary in their findings regarding the risk of postoperative infections in patients taking TNF-alpha inhibitors (Table 1) whereas prior studies suggest that methotrexate is not a substantial risk factor for surgical site infection (SSI) [19, 24].

**Table 1:** studies on the influence of biologic DMARDs on SSI rate

Author, year	Type of surgery	Number of operations	bDMARD	Main finding in patients not stopping bDMARDs
Bibbo & Goldberg, 2004 [10]	Foot and ankle surgery	31	Continued treatment perioperatively	SSI rate decreased
Talwalkar et al., 2005 [11]	Various orthopedic surgeries	11	TNF inhibitors discontinued 2–4 weeks prior to surgery or continued treatment perioperatively	SSI rate unchanged
Wendling et al., 2005 [12]	Various orthopedic surgeries	50	TNF inhibitors discontinued 2–4 weeks prior to surgery or continued treatment perioperatively	SSI rate unchanged
Giles et al., 2006 [13]	Various orthopedic surgeries	91	Continued treatment perioperatively	SSI rate increased
Broeder et al., 2007 [14]	Various orthopedic surgeries	1219	TNF inhibitors discontinued 4 t1/2 prior to surgery or continued treatment perioperatively	SSI rate unchanged
Ruyssen-Witrand et al., 2007 [15]	Various surgeries	127	Variable timing for discontinuation prior to surgery	SSI rate unchanged
Gilson et al., 2010 [16]	Total joint replacement	60	Cases treated with TNF inhibitors included	SSI rate increased
Kawakami et al., 2010 [17]	Various orthopedic surgeries	128	TNF inhibitors discontinued 2–4 weeks prior to surgery and restarted if no signs of infection	SSI rate increased
Suzuki et al., 2011 [18]	Arthroplasties	1626	Continued treatment perioperatively	SSI rate increased
Momahara et al., 2011 [19]	THA^1^ and TKA^2^	420	TNF inhibitors discontinued 2–4 weeks prior to surgery, cDMARDs continued	SSI rate increased
Berthold et al., 2013 [20]	Various orthopedic and hand surgeries	1596	TNF inhibitors discontinued 2–4 weeks prior to surgery or continued treatment perioperatively	SSI rate increased
Tada et al., 2016 [21]	Various orthopedic surgeries	332	TNF inhibitors discontinued 2–4 weeks prior to surgery, cDMARDs continued	SSI rate unchanged
Hayashi et al., 2017 [22]	THA^a^	99	Variable timing for discontinuation or no discontinuation prior to surgery	late SSI rate increased
Salt et al., 2017 [23]	THA^a^, TKA^b^ and total shoulder arthroplasty	2212	Variable timing for discontinuation or no discontinuation prior to surgery	SSI rate unchanged

^a^Total hip arthroplasty, ^b^Total knee arthroplasty

SSI, and specifically periprosthetic joint infection (PJI), is one of the most serious complications of arthroplasty and a leading cause of early revision [25, 26]. Risk factors for SSI include smoking, diabetes mellitus, obesity, a score > 2 on the American Society of Anesthesiologists (ASA) Physical Status scale, current infection, and use of steroids [23, 27–30]. Rheumatic disease has been shown to be an independent risk factor for PJI [31–34].

In 2017, the American College of Rheumatology (ACR) published guidelines for the perioperative management of anti-rheumatic medication in patients with rheumatic diseases undergoing elective total hip and total knee arthroplasty. According to these recommendations, TNF-alpha inhibitors should be withheld prior to surgery, and surgery should be planned for at the end of the dosing cycle [35].

The International Consensus Meeting (ICM) on orthopedic infections, held in Philadelphia in 2018, adopted the ACR guidelines and graded the level of evidence as moderate [36]. The Swedish Association for Rheumatology has recommendations which are consistent with the ACR guidelines [37].

Guidelines on the prevention of SSI from the World Health Organization (WHO) state that perioperative discontinuation of methotrexate has no effect on the risk of SSI, and perioperative discontinuation of TNF-alpha inhibitors might have a benefit in reducing the SSI rate. The evidence for this is, however, considered to be of very low quality, and it is stated that “considering the scarce (or absent) evidence to support discontinuation of treatment (with anti-TNFs) and even potential harm it may cause (methotrexate), such as the risk of flare-up of the underlying disease(s) associated with the suspension of therapy, immunosuppressive medication should not be discontinued to prevent SSI” [38].

Since 2000 the departments of Orthopedics and Rheumatology at Skåne University Hospital in Lund, Sweden, have performed orthopedic surgery without interrupting methotrexate treatment. In 2006, new local guidelines were introduced, and discontinuation of TNF-alpha inhibitors perioperatively in conjunction with orthopedic surgery in rheumatic patients was abolished. Data from a study conducted at our departments from 2003 to 2009 did not indicate that the perioperative use of TNF-alpha inhibitors or methotrexate was a clinically important risk factor for PJI [20].

The aim of the present study was to answer two questions: (i) What is the one-year incidence of SSI and PJI after total knee arthroplasty (TKA) and total hip arthroplasty (THA) in patients with inflammatory joint disease; and (ii) is there an association between the use of TNF-alpha inhibitors, prednisolone, or methotrexate, and SSI or PJI rates?

## Methods

This observational, nonrandomized retrospective single-center study used patient data from the departments of Rheumatology and Orthopedics at Skåne University Hospital in Lund, Sweden. The study was approved by the local ethics committee in Lund (Dnr 2016/880).

All patients above the age of 18 with an inflammatory joint disease who underwent primary TKA or THA between January 2006 and December 2015 were included in the study. Some patients had undergone more than one operation; the data analysis is based on cases (operations) and not individual patients. Information was collected from the local operation database. We included patients with an ICD-9 code for rheumatic disease of M058 or M059 (seropositive rheumatoid arthritis), M060 (seronegative rheumatoid arthritis), M069 (other rheumatoid arthritis), M073 (psoriatic and enteropathic arthropathies), or M080 (juvenile arthritis). The diagnosis was validated by crosschecking patients’ medical records. If the patient had been given an ICD-9 code for inflammatory joint disease in conjunction with another surgery and the diagnosis could be validated, the patient was included in the study, even they had been given a different diagnosis by the operating surgeon at the time of the primary TKA or THA (most often osteoarthritis). Patients with the included diagnoses are those that can be considered for treatment with bDMARDs and by not only studying patients with RA a larger number of patients could be included.

Postoperative infections are generally classified according to the U.S. Centers for Disease Control and Prevention (CDC) 1992 definition of nosocomial SSIs [39], where infections are divided into (i) superficial incisional SSI involving the skin and subcutaneous tissue, (ii) deep incisional SSI involving deep soft tissue of the incision, and (iii) organ/space SSI involving any part of the anatomy (e.g., organs or spaces) other than the incision that was opened and manipulated during the operative procedure. In this study we used a modified definition of SSI, according to the one generally used in the field of orthopedics, where” deep incisional SSI” and “organ/space SSI” are referred to together as PJI, and superficial incisional SSI is termed superficial SSI.

At least one of the following criteria was required for the diagnosis of PJI: (i) growth of identical microorganisms in at least two intraoperative cultures or a combination of preoperative aspiration and intraoperative cultures, (ii) presence of a sinus tract communicating with the prosthetic joint, (iii) presence of purulence without another known etiology surrounding the prosthetic device [40].

By definition, superficial SSI occurs within 30 days after surgery and PJI within 1 year. Patients were followed for 1 year after surgery, or until death or re-operation for a reason other than infection within 1year. With a longer follow-up time, one would expect to find a number of hematogenous infections, which was not our objective, since these cannot be related to medical treatment during the perioperative period. Patients were routinely contacted by a nurse 1 year after the operation and asked about postoperative complications such as infections. In 8 cases, data regarding one-year follow-up was missing, and the patients’ medical records were scrutinized for medical contacts which could indicate a SSI.

In 43 cases data regarding ASA and in 20 cases data regarding BMI were missing and excluded from analysis. In five respectively six cases data regarding dosing of prednisolone and methotrexate were missing and excluded from analysis.

Due to the low expected rate of SSI a much higher number of patients, than treated at our center, would have to be included in a matched cohort study to receive enough statistical power.

### Statistics

The association between infectious rate and medication was analyzed using chi- square test or Fisher’s exact test when appropriate, using SPSS Statistics 23 for Windows. The significance level was set at *p* < 0.05.

## Results

A search of the local operations database yielded 522 operations during the study period. Twenty-eight operations in 24 patients were excluded from further analysis: 5 patients diagnosed with polymyalgia rheumatica, 10 with osteoarthritis, 1 with calcium pyrophosphate arthritis, 1 with spondyloepiphyseal dysplasia, and in 7 cases data regarding rheumatic diagnosis could not be found. After exclusions, data were collected on 494 operations involving 395 individual patients. Ninety-two patients had undergone more than one operation.

Patient characteristics are described in Table 2. A majority of cases were female (76%), and the mean age at the time of surgery was 62 years (range 18–89). TKA comprised 51% of procedures (*n* = 245). A majority of cases had RA (69%), followed by juvenile idiopathic arthritis (JIA) (12%). The most frequently used DMARD was methotrexate (55.5%), followed by TNF-alpha inhibitors (31.8%). Patients taking both methotrexate and a TNF-alpha inhibitor comprised 18.2% of the sample. Details on treatment are summarized in Table 3*.*

**Table 2:** Patients characteristics**.** Values are number (percentage) unless otherwise indicated

**All**	**494**
Female	377 (76.3)
TKA^1^	254 (51.4)
THA^2^	240 (48.6)
Age, by time of surgery, mean (range)	62.4 (18–89)
**ASA**^**3**^**,** valid no 451	
ASA 1	9 (2)
ASA 2	268 (59.4)
ASA 3	172 (38.1)
ASA 4	2 (0.4)
BMI^4^, kg/m^2^, valid no 474, mean (range)	26.5 (14.9–44.6)
**Diagnosis**	
Rheumatoid arthritis^a^	341 (69)
Psoriatic arthritis	35 (7)
Spondyloarthritis incl. Ankylosing spondylitis^b^	29 (5.9)
Juvenile idiopathic arthritis^c^	59 (11.9)
Other diagnosis^d^	30 (6.1)

^1^Total knee-arthroplasty, ^2^Total hip-arthroplasty, ^3^American Society of Anesthesiologists (ASA) Physical Status, ^4^Body mass index

^a^Seropositive rheumatoid arthritis (*n* = 283), seronegative rheumatoid arthritis (*n* = 58)

^b^Ankylosing spondylitis (*n* = 21), other specified inflammatory spondylopathies (*n* = 6), inflammatory spondylopathy, unspecified (*n* = 2)

^c^juvenile arthritis (*n* = 40), juvenile arthritis with systemic onset (*n* = 7), juvenile polyarthritis (seronegative) (*n* = 6), juvenile arthritis, unspecified (*n* = 4), pauciarticular juvenile rheumatoid arthritis (*n* = 2)

^d^Inflammatory polyarthropathy (*n* = 1), polyarthritis, unspecified (*n* = 4), other specified arthritis (*n* = 4), monoarthritis, not elsewhere classified (*n* = 2), systemic lupus erythematosus, unspecified (*n* = 5), systemic lupus erythematosus with organ or system involvement (*n* = 3), adult-onset Still disease (*n* = 2), Crohn’s disease (*n* = 1), ulcerative colitis (*n* = 1), polymyositis (*n* = 1), systemic sclerosis (*n* = 2), other overlap syndrome (*n* = 1), arthritis unspecified (*n* = 1), systemic involvement of connective tissue, unspecified (*n* = 2)

**Table 3:** Exposure. Values are number (percentage) unless otherwise indicated

Prednisolone	214 (43.3)
Prednisolone, dose mg/d, mean (valid no 489)	5.5
**Number of ongoing DMARDs**^**1**^	
0	109 (22.1)
1	243 (49.2)
2	132 (26.7)
3	10 (2)
cDMARD^2^	343 (69.4)
Methotrexate	274 (55.5)
Methotrexate dose, mg/w, mean (valid no 488)	16
cDMARD^2^ other than methotrexate^a^	69 (14)
bDMARD^3^	193 (39.1)
TNF- alpha inhibitor^b^	157 (31.8)
bDMARD^3^, other than TNF- alpha inhibitor^c^	36 (7.3)
Methotrexate and prednisolone	124 (25.1)
Methotrexate and TNF- alpha inhibitor	90 (18.2)
Methotrexate and prednisolone and TNF- alpha inhibitor	40 (8.1)

^1^ Disease-modifying antirheumatic drug

^2^ Conventional disease-modifying antirheumatic drug

^3^ Biologic disease-modifying antirheumatic drug

^a^ azathioprine (*n* = 9), sulfasalazine (*n* = 30), hydroxychloroquine (*n* = 26), mycophenolate mofetil (*n* = 3) and leflunomide (*n* = 1)

^b^etanercept (*n* = 93), golimumab (*n* = 5), certolizumab (*n* = 12), infliximab (*n* = 15), adalimumab (*n* = 32)

^c^ abatacept (*n* = 6), rituximab (*n* = 16), anakinra (*n* = 5), tocilizumab (*n* = 14) One patient did bilateral THA at the same session and was treated with both anakinra and rituximab

The total incidence of SSI was 3.8% (*n* = 19). Of these, 12 were superficial SSI, yielding a rate of 2.4%. All of these infections resolved after wound debridement and/or antibiotic treatment.

There were seven cases of PJI, yielding a one-year rate of 1.4%. All cases of PJI occurred after TKA, and there was a statistically significant difference in the rate of PJI depending on operating site (*p* = 0.015). One of the patients who suffered a PJI had a hematogenous infection 11 months after surgery, but according to the design of the study this counted as an SSI.

One patient with PJI was treated with the TNF-alpha inhibitor etanercept, and 4 patients were treated with methotrexate. There was no statistically significant difference in the rate of infection between patients treated with a TNF-alpha inhibitor and those who were not (*p* = 0.44) or those treated with methotrexate (*p* = 1.0). No association could be found between PJI and prednisolone (*p* = 0.25), TNF-alpha inhibitor and methotrexate in combination (*p* = 1.0), methotrexate and prednisolone in combination (*p* = 1.0), TNF-alpha inhibitor, methotrexate and prednisolone in combination (*p* = 1.0), BMI (*p* = 0.19), or ASA-score (*p* = 0.44) (Table 4*).* No correlation could be found between diagnosis and the rate of PJI or the total number of SSI. No correlation could be found between the total number of SSI and treatment with prednisolone, methotrexate, TNF-alpha inhibitors or a combination of these treatments (Table 4*)*.

**Table 4:** Periprosthetic joint infection (PJI) and all surgical site infections (SSI) in various subgroups

	Total (n)	PJI (n)	*p*-value	all SSI (n)	*p*-value
Female	377	3		14	
Male	117	4	0.06^a^	5	0.78^b^
**Procedure**					
TKA^1^	254	7		11	
THA^2^	240	0	0.015^a^	8	0.33^b^
**BMI**^**3**^**,** valid no 474					
< 30	368	4		12	
> 30	106	3	0.19^a^	7	0.16 ^b^
**ASA**^**4**^**,** valid no 451					
< 2	277	3		10	
> 3	174	4	0.44^a^	8	0.60 ^b^
**Treatment**					
Methotrexate	274	4	1.0^a^	12	0.49^b^
TNF-alpha inhibitor	157	1	0.44^a^	5	0.60^b^
Prednisolone	214	5	0.25^a^	10	0.40^b^
Methotrexate and prednisolone	124	2	1.00^a^	4	0.79^a^
Methotrexate and TNF-inhibitor	90	1	1.00^a^	3	1.00^a^
Methotrexate, TNF- inhibitor and prednisolone	39	0	1.00^a^	0	0.39 ^a^
**Diagnosis**					
Rheumatoid arthritis	341	6	0.45^a^	16	0.21^a^
Psoriatic arthritis	35	1	0.40^a^	2	0.64^a^
Spondyloarthritis incl. Ankylosing spondylitis	29	0	1.0^a^	0	1.0^a^
Juvenile idiopathic arthritis	59	0	1.0^a^	1	0.76^a^
Other diagnosis	30	0	1.0^a^	0	1.0^a^

^1^Total knee-arthroplasty, ^2^Total hip-arthroplasty, ^3^Body mass index, ^4^American Society of Anesthesiologists (ASA) Physical Status

^a^Fisher’s exact test

^b^Chi-square test

Five out of 7 PJI healed after treatment with debridement and antibiotics. Details on patients suffering PJI, including outcome are described in Table 5.

**Table 5:** Periprosthetic joint infection (PJI), individual cases^a^

Diagnosis	Type of surgery	Anti-rheumatic treatment	Infectious agents	Treatment of PJI	Outcome
RA^b^, seronegative	TKA^c^	Methotrexate, prednisolone	*S. aureus*	Debridement and exchange of tibial insert	Healed (26 months later re-infected with the same bacteria)
RA^b^, seropositive	TKA^c^	Etanercept, methotrexate	coagulase negative staphylococcus (CNS)	Two-stage revision	Healed
RA^b^, seropositive	TKA^c^	Prednisolone, azathioprine	*S. aureus*	Debridement and exchange of tibial insert	Failure (chronic infection treated with suppressive antibiotics)
RA^b^, seropositive	TKA^c^	Methotrexate, prednisolone	*B. fragilis*	Antibiotics	Failure, amputation
RA^b^, seropositive	TKA^c^	Methotrexate, sulfasalazine, hydroxychloroquine, prednisolone	*S. mitis, S. hominis*	Debridement and exchange of tibial insert	Healed
RA^b^, seropositive	TKA^c^	Prednisolone	coagulase negative staphylococcus (CNS)	Debridement and exchange of tibial insert	Healed
PsA^c^	TKA^c^	None	coagulase negative staphylococcus (CNS)	Debridement and exchange of tibial insert	Healed

^a^In order to not compromise patient confidentiality age and sex is excluded from the table. There were 4 males and 3 females and the mean age was 64 years (range 44–70)

^b^Rheumatoid arthritis

^c^psoriatic arthritis

Four out of seven of patients with a PJI were male, although only 24% of the operations were performed on male patients. However, there was no statistically significant difference in the rate of PJI between men and women (*p* = 0.06) (Table 4*)*.

Six patients died within 1 year of surgery*.* One patient died 20 days after surgery due to a gastrointestinal bleeding. Three patients died due to acute coronary syndrome, one due to a subarachnoid hemorrhage and one due to progressive dementia (Pick’s disease). None of the deaths within 1 year of surgery could be linked directly to surgery or PJI.

Four patients underwent reoperation within 1 year of surgery. One patient was reoperated due to joint instability, two due to aseptic loosening of the prosthesis, and one patient due to fracture after a resurfacing hip arthroplasty.

## Discussion

The main finding in this study is that among patients with inflammatory joint disease undergoing primary knee or hip arthroplasty no association could be found between and PJI, or SSI in general and continued treatment with TNF-alpha inhibitors. Neither was there any association between infection rate and treatment with prednisolone, methotrexate, or combinations of treatments.

A previous study at our center [20] showed an increased risk of SSI in orthopedic surgery in general amongst patients who continued treatment with TNF-alpha inhibitors perioperatively compared to patients discontinuing medication; however, the finding was due to a very low incidence rate of SSI following foot surgery in the comparison group.

A meta analysis comparing continuation versus discontinuation of TNF-alpha inhibitors prior to orthopedic surgery favors discontinuation of bDMARDs [8], although the studies included are heterogeneous in terms of differing timing of treatment interruption and the variety of patients and operations included. Furthermore, the included studies were underpowered to detect small changes in infection rate.

As shown in Table 1*,* other studies on the influence of TNF-alpha inhibitors on infection rates comes to different conclusions. Seven studies showed a slight increase in PJI rate [13, 16–20, 22], while seven [10–12, 14, 15, 21, 23] showed no increase or a decrease in PJI in patients treated perioperatively with TNF-alpha inhibitors. The studies included different types of surgery, some compared patients not treated with TNF-alpha inhibitors to those treated with TNF-alpha inhibitors, and some compared the results of patients who continued TNF-alpha inhibitors prior to surgery versus those who discontinued. Furthermore, the timing of treatment interruption varied, all of which makes conclusions difficult.

The rate of PJI was 1.4% which is comparable to what have been found in other studies on subjects with rheumatic disease [31, 41].

The rate of PJI after TKA was 2.8%. This is somewhat higher than the one-year incidence for revision due to infection of 1.7% for TKAs performed between 2006 and 2011reported from the Swedish Knee Arthroplasty Register (SKAR) [42]. The majority of patients in SKAR have osteoarthritis, and patients with RA have previously been shown to have a increased risk for PJI compared to patients with osteoarthritis [41, 43].

None of the THAs performed in our study resulted in a PJI. The Swedish Hip Arthroplasty Register (SHPR) reported an re-operation rate due to infection of 1.2% following THA performed in Sweden between 2005 and 2008 [44]. The fact that the incidence rate of THA was lower than expected could be coincidental or it could be due to low number of procedures in this study, although the same pattern - a higher risk of revision in RA patients undergoing a TKA than a THA - has been showed previously [43].

In only one of the seven cases of PJI was the patient treated with a TNF-alpha inhibitor: in this case, etanercept in combination with methotrexate. In total, 157 patients were treated with a TNF-alpha inhibitor (31.8%), which means that only 0.63% of patients treated with a TNF-alpha inhibitor suffered a PJI.

From 2003 to 2005, before the new policy of perioperative continuation of TNF-alpha inhibitors was introduced in our departments, only 0.6% of implant operations led to a PJI [20]. The present finding of 1.4% is higher than this very low incidence rate, but the very small number of cases makes interpretation difficult.

One patient with PJI suffered a hematogenous infection with *Streptococcus mites* 11 months after surgery. The fact that the infection occurred so long after surgery may suggest that the treatment at the time of surgery was not a significant contributor to the infectious outcome. Leaving out this infection, the total rate of PJI would have been 1.2%.

Men were overrepresented in the group with PJI (*p* = 0.06), which is consistent with previous findings [23, 28, 29].

PJI is a serious complication of arthroplasty, and potential risk factors for infection should be eliminated prior to surgery whenever possible. With this in mind, the risk of flares in patients with rheumatic disease when discontinuing DMARD treatment should be carefully considered when deciding whether to continue or discontinue DMARD treatment prior to surgery. Although our center does not perioperatively discontinue TNF-alpha inhibitors prior to elective arthroplasty surgery, we do not apply this approach as a general rule for others DMARDs such as rituximab, tocilizumab, or JAK inhibitors, for which data is scarcer.

This is a single-center study, and the procedures were performed by a few experienced surgeons following the same routines throughout the study period. One-year follow-up was standardized via a telephone call, and data on perioperative medications were retrieved by one investigator (YB) using medical records. There was little missing data (Table 3), mostly regarding dosing of methotrexate and prednisolone, which did not affect the result.

The limitations of this study include its observational and descriptive nature, insofar as it did not include a control group pausing treatment with bDMARDs. The overall rate of SSI, including PJI, was low, and it is thus underpowered, as are all other similar investigations. Based on the available data, presented in Table 1, it seems likely that treatment with TNF-alpha inhibitors per se confers a somewhat increased risk of PJI. The importance of stopping treatment perioperative is unclear. In fact, it is not likely that a randomized, controlled trial of stopping bDMARDs perioperatively will be performed, as this would require a very large number of procedures. Assuming a general PJI incidence of 2%, a clinical trial would have to include more than 21,000 procedures within each group (continuation versus cessation of bDMARD perioperatively) to detect a 20% increase of PJI incidence with a power of 80%. Furthermore, each bDMARD would require its own trial.

## Conclusions

We found no signs of increased PJI risk despite perioperative DMARD treatment in patients with inflammatory joint disease undergoing elective TKA or THA. Our center’s policy of perioperative continuation of TNF-alpha inhibitors and most other bDMARDs will remain unchanged. Larger, preferably multi-center, studies are needed to elucidate the role, if any, of perioperative treatment cessation in reducing PJI rates.

## Data Availability

The datasets used and/or analysed during the current study will be available from the corresponding author on reasonable request.

## Abbreviations

ACR: American College of Rheumatology
ASA: American Society of Anesthesiologists (ASA) Physical Status
bDMARD: Biologic disease-modifying antirheumatic drug
cDMARD: Conventional disease-modifying antirheumatic drug
CNS: Coagulase negative staphylococcus
ICD-9: International Statistical Classification of Disease
ICM: International Consensus Meeting
PJI: Periprosthetic joint infection
PsA: Psoriatic arthritis
RA: Rheumatoid arthritis
SHPR: Swedish Hip Arthroplasty Register
SKAR: Swedish Knee Arthroplasty Register
SRF: Svensk reumatologisk förening
SSI: Surgical site infection
THA: Total hip- arthroplasty
TKA: Total knee- arthroplasty
TNFα: Tumor necrosis factor alpha
WHO: World Health Organisation
